# Cation Pretreatment Enables the Saline Stability of a Near-Infrared Sensor for Dopamine

**DOI:** 10.1021/acsbiomedchemau.4c00094

**Published:** 2025-01-27

**Authors:** Xuewen Liu, Jing Chen, Hanxuan Wang, Benjamin Lambert, Ardemis A. Boghossian

**Affiliations:** † Henan Agricultural University, Zhengzhou, Henan 450002, China; ‡ Ecole Polytechnique Fédérale de Lausanne (EPFL), 1015 Lausanne, Switzerland; § Université de Bordeaux, LP2N - Institut d’Optique, CNRS, F-33405 Talence, France

**Keywords:** single-walled carbon nanotubes (SWCNTs, SWNTs), near-infrared fluorescence, dopamine sensor, deoxyribose nucleic acid (DNA), phosphate backbone, trivalent cations

## Abstract

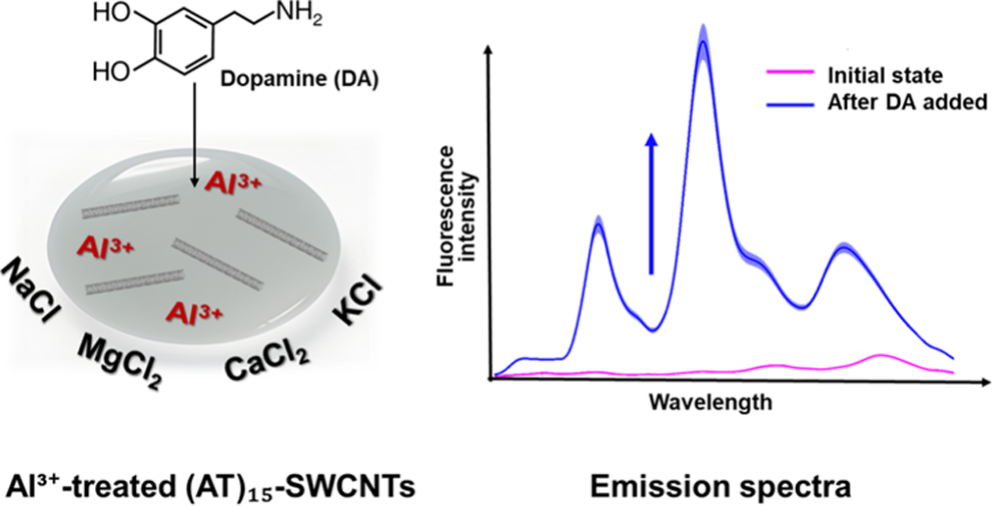

Single-walled carbon nanotubes (SWCNTs) are wrapped with single-stranded DNA (ssDNA) to create near-infrared (NIR-II) fluorescent sensors for diverse analytes. However, the interaction between the negatively charged backbone of ssDNA and cations in biological saline alters fluorescence unpredictably. This susceptibility limits the application of these sensors in biological media. To address this limitation, this study develops a cation-pretreatment strategy that quenches the baseline fluorescence of ssDNA-SWCNTs to enable turn-on responses that are selectively triggered by analytes in saline. An initial screening of Na^+^, K^+^, Mg^2+^, Ca^2+^, and Al^3+^ pretreatments of gel-encapsulated (AT)_15_-SWCNTs reveals that Al^3+^ pretreatment induces a stable quenching of fluorescence that is reversible only on Al^3+^ chelation or precipitation. We apply this Al^3+^ pretreatment to develop a saline-resilient, near-infrared sensor for dopamine. The Al^3+^-treated (AT)_15_-SWCNTs show a concentration- and chirality-dependent fluorescence response over a dynamic range of 1 nM and 10 μM dopamine, achieving a 110-fold increase in the turn-on response to 10 mM dopamine in buffered saline compared with the untreated (AT)_15_-SWCNTs. Further study of the effects of pH and different salts on the dopamine response suggests a mechanism that relies on competing trivalent cations and negative DNA phosphate interactions. These interactions lay the framework for saline-resilient optical sensors that exploit DNA as a charged-based actuator for modulating the exciton dynamics and controlling the SWCNT fluorescence.

## Introduction

Single-walled carbon nanotubes (SWCNTs) emit fluorescence in the near-infrared range, which is ideal for optical sensing. This fluorescence stems from the radiative recombination of excitons diffusing across the surface of the SWCNT. With every carbon of the SWCNT exposed as a surface atom, the fluorescence can respond strongly to even the slightest changes in the SWCNT environment. The surfaces of SWCNTs are thus often modified to control these changes and, consequently, the optical response to different analytes. These responses stem from different mechanisms that include charge transfer, changes in the chemical skeleton or configuration of the surface modifications, and/or alterations of other factors such as pH, local dielectric, and water accessibility to the surface.^1,2^ Single-stranded DNA (ssDNA) is often used to modify the SWCNT surface, as pi-pi stacking of its bases on the surface provides intimate control over the exciton dynamics. Importantly, the different sequences of ssDNA show distinct fluorescence responses to different bioanalytes, including glucose,^3^ nitric oxide,^4^ lipids,^5^ and neurotransmitters such as dopamine,^6^ serotonin,^7^ and norepinephrine.^8^ Specific sequences of ssDNA-SWCNTs have also been used to monitor the release of drugs including chemotherapeutic agents.^9,10^ This sequence specificity allows the sensor response to be tuned simply by alteration of the DNA sequence. Additionally, ssDNA-SWCNTs benefit from low detection limits, biotransparency, and photostability that further advance applications in noninvasive *in vivo* and continuous monitoring.

However, such biological applications require measurements in complex fluids and saline that contain cations. The cations can interact with ssDNA, which can change the DNA conformation and the SWCNT fluorescence. The cations in biofluids can even precipitate ssDNA-SWCNTs, quenching their fluorescence.^2^ The mechanisms underlying these effects may stem from differences in the DNA wrapping conformation,^3^ which can affect the SWCNT interactions with water,^4^ oxygen,^11^ or the ssDNA itself.^12^ For example, saline environments have been shown to increase the DNA coverage of SWCNTs by screening or neutralizing the negatively charged phosphate groups of the DNA. This increased coverage leads to increased water exclusion and oxygen exclusion, which can alter the SWCNT fluorescence.^2,11,13,14^ Since optical SWCNT sensors rely on intensity and/or wavelength shifting responses,^15^ the saline can thus interfere with the desired response to the bioanalyte.

Mitigating saline effects is therefore crucial for enhancing the sensor performance in complex environments. Previous studies have reduced the interfering effect of salts on fluorescence by introducing locked nucleic acid (LNA) nucleotides at certain positions along the oligonucleotide wrapping.^16^ The LNA nucleotides are RNA bases modified with a covalent bridge between the 2′ oxygen and 4′ carbon on the ribose moiety. This bridge increases the rigidity of the oligonucleotide backbone by restraining the wrapping into the 3′-endo (north) conformation. Compared to unmodified ssDNA-SWCNTs, the corresponding LNA-based SWCNT sensor showed increased resilience against the wavelength shifting caused by ions. The positions of the LNA nucleotides in the wrapping, however, were chosen randomly, and subsequent work showed the fluorescence of the SWCNT to depend strongly on the LNA positioning.^17^ Furthermore, LNA is significantly more costly than DNA, limiting the number of configurations that can be explored when these sensors.

Compared with LNA, cation treatment offers a more accessible means of stabilizing DNA on SWCNTs. Such treatments take advantage of the electrostatic interaction of the cation with the negative DNA backbone. For certain trivalent cations, the interaction may further trigger a counterion condensation reaction that collapses double-stranded DNA into a compact state.^18,19^ The compact state shows more restricted degrees of freedom that may limit nonspecific conformational perturbations from interfering ions.

Herein, we report a strategy based on the chelation of cations to minimize the effect of nonspecific saline interactions on the fluorescence of ssDNA-SWCNTs. In particular, we examined the effect of the chelation on the response of ssDNA-wrapped SWCNTs to dopamine. Previous studies have explored dopamine detection through the use of double-stranded DNA^20^ and the application of cation-based strategies.^21^ In this study, we aim to minimize interfering salt effects for ssDNA-SWCNT sensors based on a cation-pretreatment strategy that is accessible and compatible with diverse DNA sequences. This study thus demonstrates a previously unexplored avenue for stabilizing optical SWCNT-based sensors for measurements *in vivo* and in complex biofluids.

## Results and Discussion

**Figure 1:**
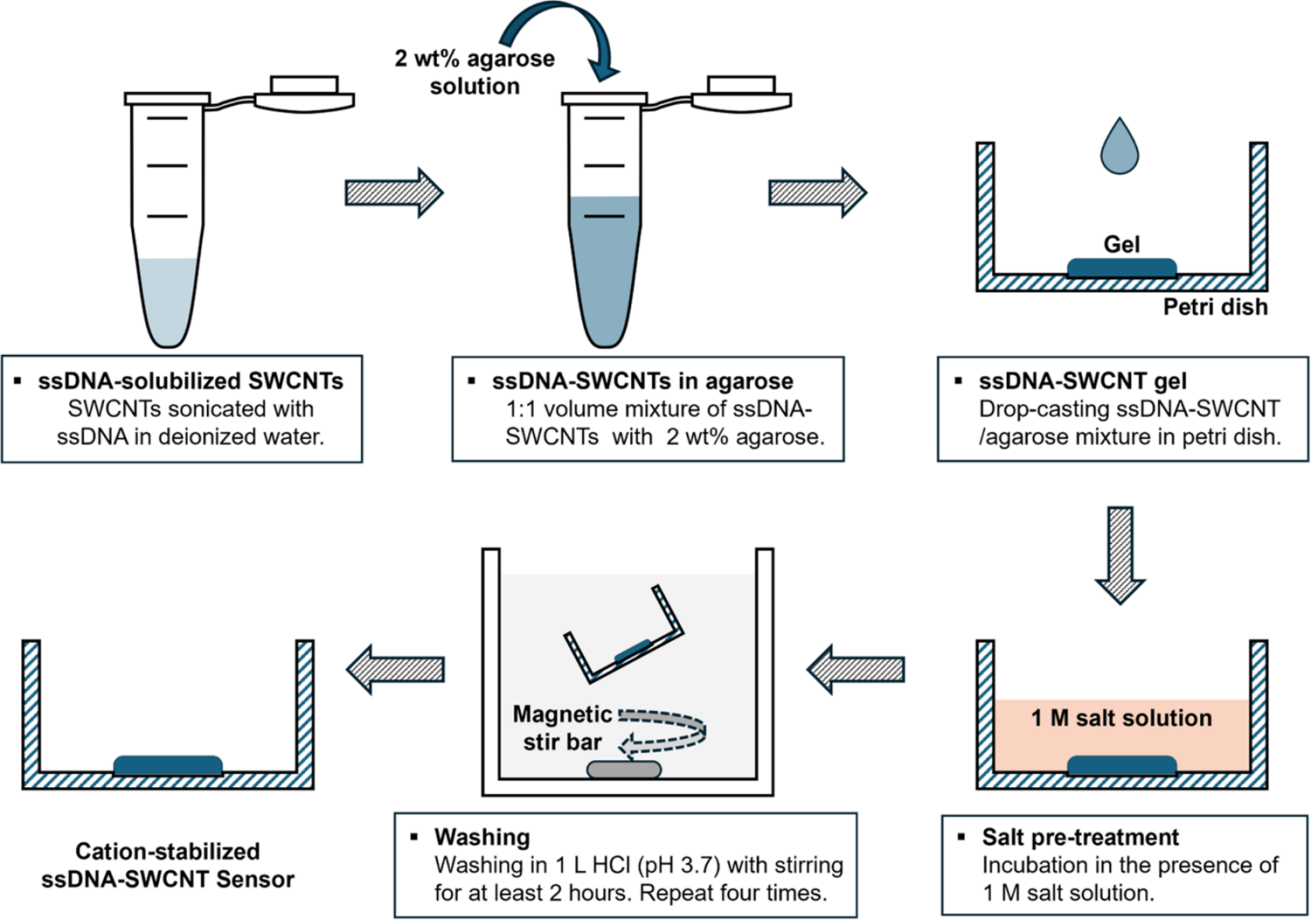
Preparation of gel-based cation-stabilized (AT)_15_-SWCNT sensors. The SWCNTs are solubilized by ssDNA through sonication (top left). The ssDNA-SWCNT solution is mixed with 2 wt % agarose solution in a 1:1 volume ratio (top center), and 190 μL of the ssDNA-SWCNT/agarose mixture is drop-casted onto a glass-bottomed Petri dish (top right). After drying, the gel is incubated in the presence of 1 M salt solution for 2 h (bottom right). The gel is washed by incubating in 1 L of HCl (pH 3.7) for at least 2 h under mixing (bottom center). The washing step is repeated four times before the final cation-stabilized ssDNA-SWCNT sensor (bottom left) is obtained.

The preparation of the sensors is summarized in Figure 1 (see also the protocol in Supporting Information). The SWCNTs are wrapped with either the (GT)_15_ or (AT)_15_ ssDNA sequence, sequences that have been previously reported to elicit a SWCNT fluorescence response to dopamine,^6,22^ and the fluorescence is monitored for the (7,6) and (9,4) SWCNT chiralities, the dominant chiralities in the SWCNT mixture. The final construct, shown at the bottom left of Figure 1, consists of gel-encapsulated ssDNA-SWCNTs immobilized onto the bottom of a Petri dish.

**Figure 2:**
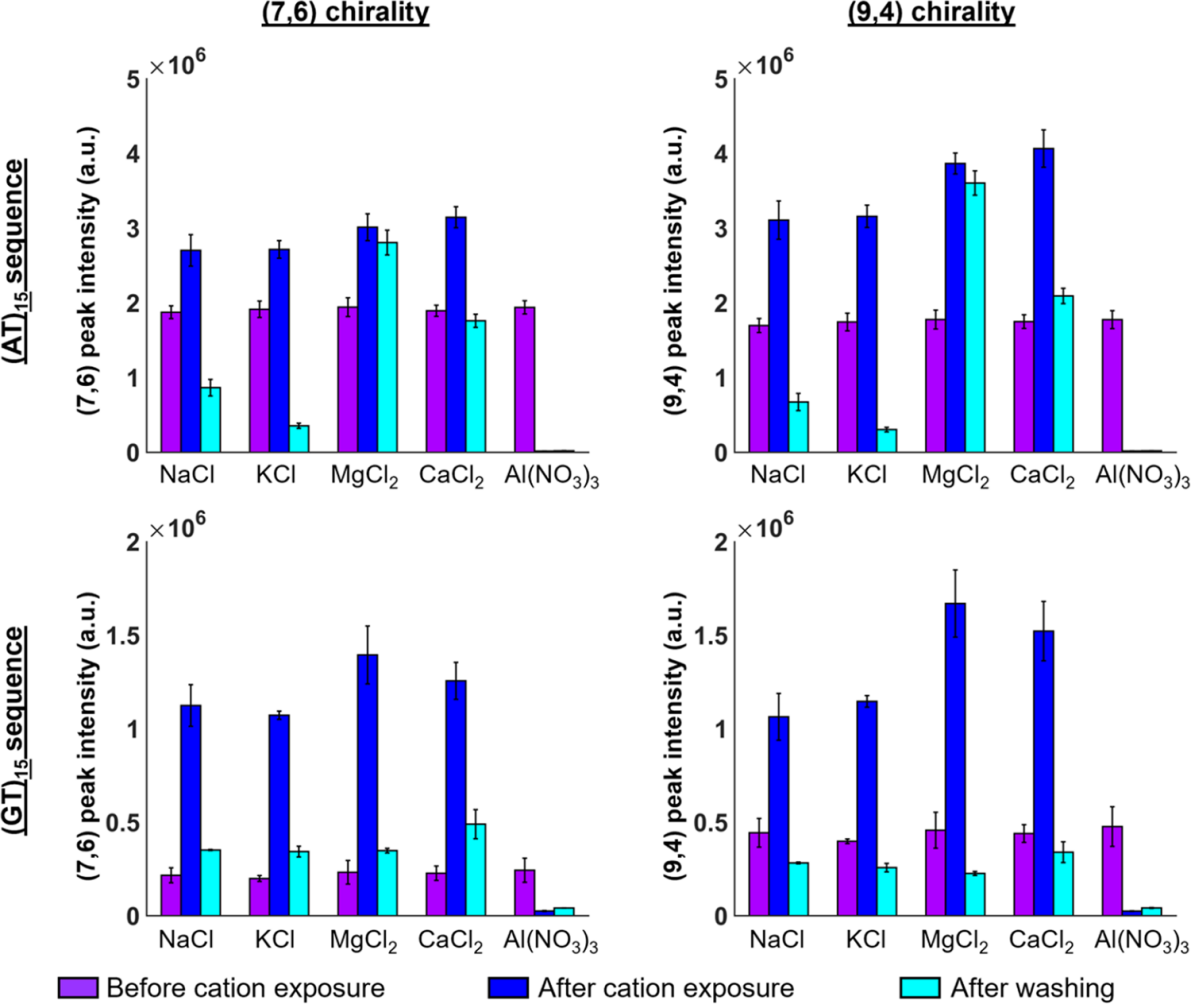
Fluorescence intensity of ssDNA-SWCNTs after pretreatment with different cations. Fluorescence intensity of gel-encapsulated ssDNA-SWCNTs before cation incubation (violet), after 1 M cation incubation (blue), and after washing (cyan). Intensities are shown for the (7,6) and (9,4) chirality (right) for SWCNTs wrapped with the (AT)_15_ ssDNA sequence (top) and the (GT)_15_ ssDNA sequence (bottom). Samples were excited at 655 and 730 nm excitation for the (7,6) and (9,4) chiralities, respectively, and emission intensities were measured at the corresponding chirality peak maximum. Error bars represent the standard deviation of the three replicates.

To examine the effects of different cation pretreatments on the sensor response, the freshly prepared SWCNT gels were incubated with 1 M solutions of NaCl, KCl, MgCl_2_, CaCl_2_, or Al(NO_3_)_3_. As shown in Figure 2, the ssDNA-SWCNTs show an increase in fluorescence intensity on the addition of the monovalent salts, NaCl and KCl, for both the (7,6) and (9,4) chiralities of the (AT)_15_ and (GT)_15_ ssDNA-wrapped SWCNTs. These observations are consistent with previous studies that have shown an increase in SWCNT fluorescence in the presence of ions that favor tighter DNA wrapping conformations on the SWCNT.^2,16^ This enhancement, however, is diminished in all cases after washing. Furthermore, except for the (7,6) chirality of the (GT)_15_-SWCNTs, the intensities after acid washing decrease to values below those from before the addition of the cations. These changes may reflect a change in the DNA conformation and/or pH-induced fluorescence quenching.^23^

Similarly, the divalent salts CaCl_2_ and MgCl_2_ increase the fluorescence of the (GT)_15_-SWCNTs and (AT)_15_-SWCNTs (Figure 2). These observations contrast with previous studies that have reported the Stern–Volmer quenching of surfactant-suspended SWCNTs in the presence of Ca^2+^ and Mg^2+^ cations.^24^ The fluorescence enhancement is thus attributed to divalent cation-induced ssDNA conformational changes that have been reported previously to enhance the fluorescence of gel-immobilized ssDNA-SWCNTs under certain conditions.^16^ For the CaCl_2_-incubated sensors, both the (7,6) and (9,4) chiralities of the (GT)_15_-SWCNTs and (AT)_15_-SWCNTs decrease in fluorescence after washing. Whereas the MgCl_2_-incubated (GT)_15_-SWCNTs similarly decrease in fluorescence, the (AT)_15_-SWCNTs interestingly show no significant change in fluorescence for either the (7,6) or (9,4) chiralities. This difference is attributed to adenine-specific Mg^2+^ interactions that may strengthen the Mg^2+^ binding to the (AT)_15_ sequence.^25^

Compared to the monovalent and divalent cations, the trivalent Al^3+^ cation quenches both the (7,6) and (9,4) chiralities of the (AT)_15_-SWCNT and (GT)_15_-SWCNTs (Figures 2 and S1). These findings appear to contrast with previous works that have reported negligible quenching for surfactant-suspended SWCNTs in the presence of Al^3+^.^24^ Though this previous work was limited to more moderate metal concentrations of up to 5 mM per 15 mg/L SWCNTs, it also reported a relatively low charge density and Stern–Volmer quenching constant for Al^3+^. These findings suggest that the quenching observed herein is unlikely to be due to direct metal quenching of the SWCNT by Al^3+^; instead, it likely reflects a mechanism that is DNA–dependent. This hypothesis is consistent with previous reports on the complexation of Al^3+^ with the oxygen atoms of the DNA phosphate groups.^26,27^ Furthermore, the quenching is sustained even after washing, reflecting the stronger specific binding affinity that would be expected for the trivalent cation.^28^

**Figure 3:**
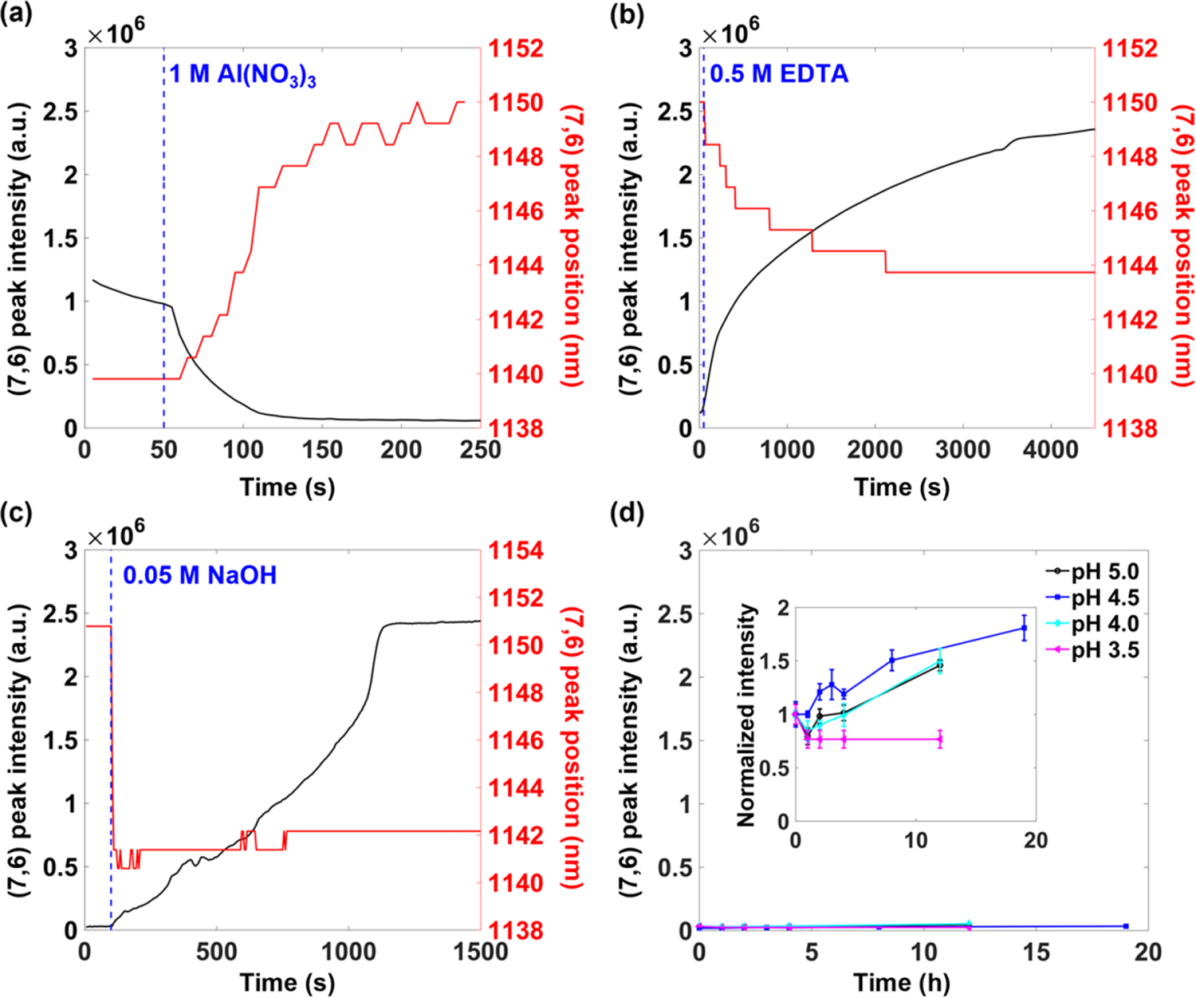
Kinetic response and reversibility of Al^3+^ pretreatment. (a) Fluorescence intensity (black) and peak wavelength (red) of (7,6) gel-encapsulated (AT)_15_-SWCNTs over time following the addition of 1 M Al(NO_3_)_3_. After Al(NO_3_)_3_ pretreatment, the fluorescence intensity (black) and peak wavelength (red) of the (7,6) gel-encapsulated Al^3+^-(AT)_15_-SWCNTs were monitored following (b) 0.5 M EDTA addition and (c) 0.05 M NaOH addition. The blue vertical dotted lines correspond to the time at which Al(NO_3_)_3_, EDTA, and NaOH were added in panels (a), (b), and (c), respectively. (d) Fluorescence intensity of (7,6) gel-encapsulated Al^3+^-(AT)_15_-SWCNTs over time at different acidic pHs. Samples were immersed at the corresponding pH at time = 0. The inset represents the same plot based on intensity normalized by the initial value. Error bars represent the standard deviation of three replicates. Samples were excited at 655 nm, and emission intensities were measured at the (7,6) chirality peak maximum.

The kinetics of the ssDNA-SWCNT interaction with Al^3+^ was further studied through continuous fluorescence monitoring of the (7,6) chirality of the (AT)_15_-SWCNTs (Figure 3). As shown in Figure 3a, the maximum peak intensity diminishes almost completely on 1 M Al(NO_3_)_3_ addition, consistent with the observations in Figure 2. This decrease is accompanied by a concomitant 10 nm red-shifting of the fluorescence peak. Both the quenching and shifting effects also appear for other SWCNT chiralities in the presence of Al^3+^ (Figure S2). The shifting is consistent with previous studies that have shown fluorescence red-shifting from monovalent and divalent cation-induced changes in DNA conformation.^14,16,29,30^ While the red-shifting may be attributed to increased water accessibility to the surface of the SWCNT, previous studies have also demonstrated red-shifting through electron withdrawal from the SWCNT.^31−35^ Electron withdrawal has also been shown in previous studies to quench SWCNT fluorescence, particularly in the presence of strong oxidizing agents.^36−38^ The concomitant fluorescence quenching and red-shifting therefore suggest a mechanism based on electron withdrawal. Such a mechanism is consistent with Al^3+^ complexation of the negatively charged phosphate groups on the SWCNT surface.

The reversibility of this change was monitored through the addition of a metal chelating agent (Figure 3b) and a base (Figure 3c). The metal chelating agent ethylenediaminetetraacetic acid (EDTA) (Figure 3b) partially reverses the shifting and recovers the fluorescence intensity to values exceeding the initial intensities. This fluorescence enhancement is attributed to the Na^+^ in the EDTA solution, which was previously reported to increase the ssDNA-SWCNT fluorescence in the chelation of divalent cations.^2,16^ The reversibility was also observed with the addition of 0.05 M NaOH (Figure 3c). However, compared to the EDTA-exposed sensors, the NaOH-exposed sensors show distinct kinetics, undergoing an immediate blue-shifting and linear increase in fluorescence compared to the gradual shifting and Langmuir-like increase that is observed with EDTA. These kinetics suggest a distinct pH-dependent mechanism for the recovery of the fluorescence.

The pH and cationic dependence of the fluorescence recovery was further studied by exposing the Al^3+^-treated (AT)_15_-SWCNTs to different pH values (Figure 3d). As shown in the figure, the Al^3+^-treated SWCNTs show limited intensity responses to pH, with the fluorescence remaining quenched. A comparison of normalized intensity over 10 h, however, shows slight pH-specific variations (Figure 3d, inset). Exposure to an acidic pH of 3.5, comparable to the pH of the washing solution, shows no substantial change in fluorescence, whereas exposure to a higher pH of 4.0 results in a slight linear increase in fluorescence. Exposure to an even higher pH of 4.5, however, yields a diminished fluorescence increase. The larger fluorescence recovery is observed only on exposure to an even higher pH of 5.0. These observations contrast with previous studies that have reported a monotonic increase in SWCNT fluorescence with increasing pH,^23^ suggesting a mechanism beyond the direct pH modulation of the SWCNT fluorescence.

In addition to SWCNT fluorescence, the pH affects the speciation of Al^3+^.^39^ Considering only mononuclear speciation (Al_n_ (OH)_x_ where *n* = 1), the Al^3+^ undergoes the hydrolysis reactions summarized below^40,41^


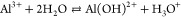
reaction 1
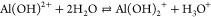
reaction 2
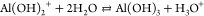
reaction 3
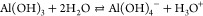
reaction 4
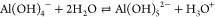
reaction 5Though salts such as Al(NO_3_)_3_ are soluble in water, the hydrolysis product, Al(OH)_3_, formed from the Al^3+^ dissociation is relatively insoluble. The formation of this product can therefore effectively remove soluble Al^3+^ from the solution. For the dissociation reaction
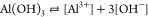
reaction 6the corresponding solubility product constant, *K*_SP_, is
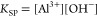
1For insoluble Al(OH)_3_ in the presence of acid, the excess [H^+^] under acidic conditions can react with the [OH^–^] anion
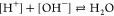
reaction 7thereby decreasing the reaction quotient, *Q*
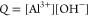
2Accordingly, the Al(OH)_3_ will continue to dissolve until *Q* = KSP based on Le Chatelier’s principle. The addition of acid therefore favors the solubilization of Al^3+^. Exposure to basic conditions would thus remove Al^3+^ through the preferential formation of hydroxide products. Assuming approximately^5^ Al^3+^ cations per (AT)_15_ sequence^42^ and 170 adsorbed sequences per SWCNT,^43^ the Al^3+^-treated (AT)_15_-SWCNT gel contains an effective Al^3+^ concentration of 4.30 μM (see calculation in Supporting Information). Under these conditions, the experimental phase diagram shows the favorable solubilization of Al^3+^ for pHs below approximately pH 5.2.^44^ This favorable solubilization is largely consistent with the transition in the fluorescence response that is observed between pH 4 and pH 5 in Figure 3d. It is also consistent with previous studies that report a relative decrease in Al^3+^ concentrations above pH 4 and the preferential formation of Al hydrolysis products above pH 5.^41^ The trend in Figure 3d thus reflects a trade-off between the increasing fluorescence effects of pH on SWCNTs, oxygen exclusion from Na^+^-induced conformation changes, and effective Al^3+^ removal through hydrolysis and the decreasing fluorescence effect from Na^+^-induced tighter wrapping conformations that disfavor the removal of the quenching cations.

**Figure 4:**
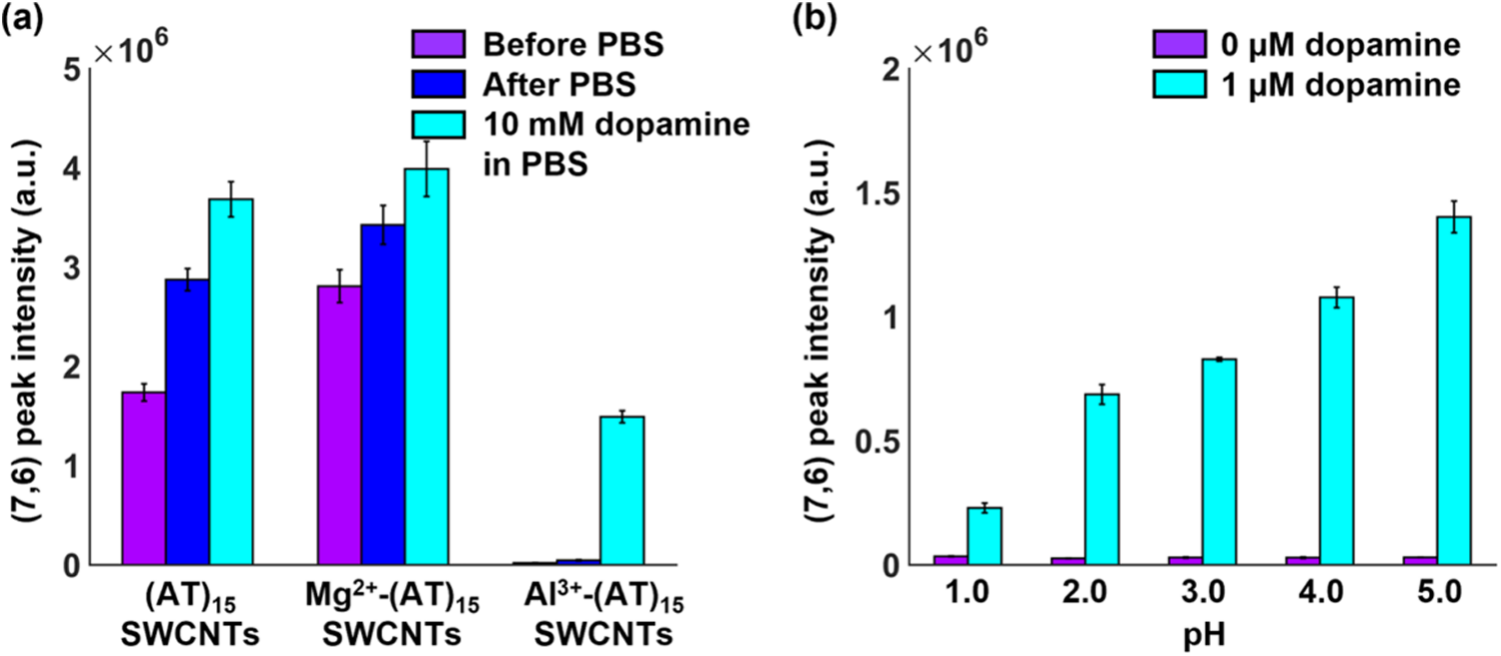
Effects of pretreatment and pH on dopamine response of (AT)15-SWCNTs. (a) Fluorescence intensity of (7,6) gel-encapsulated (AT)_15_-SWCNTs in the absence of buffer (violet), in the presence of PBS buffer (blue), and after 10 mM dopamine addition in the presence of buffer (cyan). Measurements were taken for untreated (AT)_15_-SWCNTs ((AT)_15_-SWCNTs, left), (AT)_15_-SWCNTs pretreated with 1 M MgCl_2_ (Mg^2+^-(AT)_15_-SWCNTs, center), and (AT)_15_-SWCNTs pretreated with 1 M Al(NO_3_)_3_ (Al^3+^-(AT)_15_-SWCNTs, right). (b) Fluorescence intensity of (7,6) gel-encapsulated Al^3+^-pretreated (AT)_15_-SWCNTs at different pHs in the absence (violet) and presence (blue) of 1 μM dopamine.

Since both the MgCl_2_- and Al(NO_3_)_3_-incubated (AT)_15_-SWCNTs undergo negligible intensity changes after washing, their fluorescence stability and response to dopamine was monitored in the presence of phosphate buffer saline (PBS) (Figures 4a and S3a). The untreated and MgCl_2_-pretreated sensors show an initial increase in fluorescence in PBS followed by 28 and 16% increases in the presence of 10 mM dopamine, respectively. This increase in dopamine addition is consistent with previous observations that attribute the fluorescence increase to an increase in the exciton diffusion and a decrease in the nonradiative decay rate constant from dopamine binding.^45^ These effects are hypothesized to arise from the interaction of the DNA phosphate backbone with the OH groups of the dopamine, which brings the DNA closer to the SWCNT surface to effectively reduce exciton quenching.^46^ In contrast to the MgCl_2_-pretreated sensor, the Al(NO_3_)_3_-pretreated sensor shows no significant response to PBS. In addition, while the MgCl_2_-pretreated sensor shows a lower response to dopamine compared with the untreated (AT)_15_-SWCNT sensor, the Al(NO_3_)_3_-pretreated sensors show a 110-fold higher relative response to dopamine compared with the untreated sensor.

The dependence of this response on pH was studied by exposing the Al^3+^-treated (AT)_15_-SWCNTs to 1 μM dopamine, the maximum dopamine concentration after neuron bursting,^47^ at different pHs (Figures 4b and S3b). The measurements were taken for pH < 5 to minimize the precipitation of Al(OH)_3_.^41^ The Al^3+^-treated (AT)_15_-SWCNTs remain quenched across all pH values, consistent with the observations of Figure 3d. As with PBS, the fluorescence increases in the presence of dopamine, albeit with an increase in response to increasing pH. These findings are consistent with previous reports that have shown an enhanced fluorescence response to dopamine for (GT)_6_-SWCNTs with increasing pH.^48^

**Figure 5:**
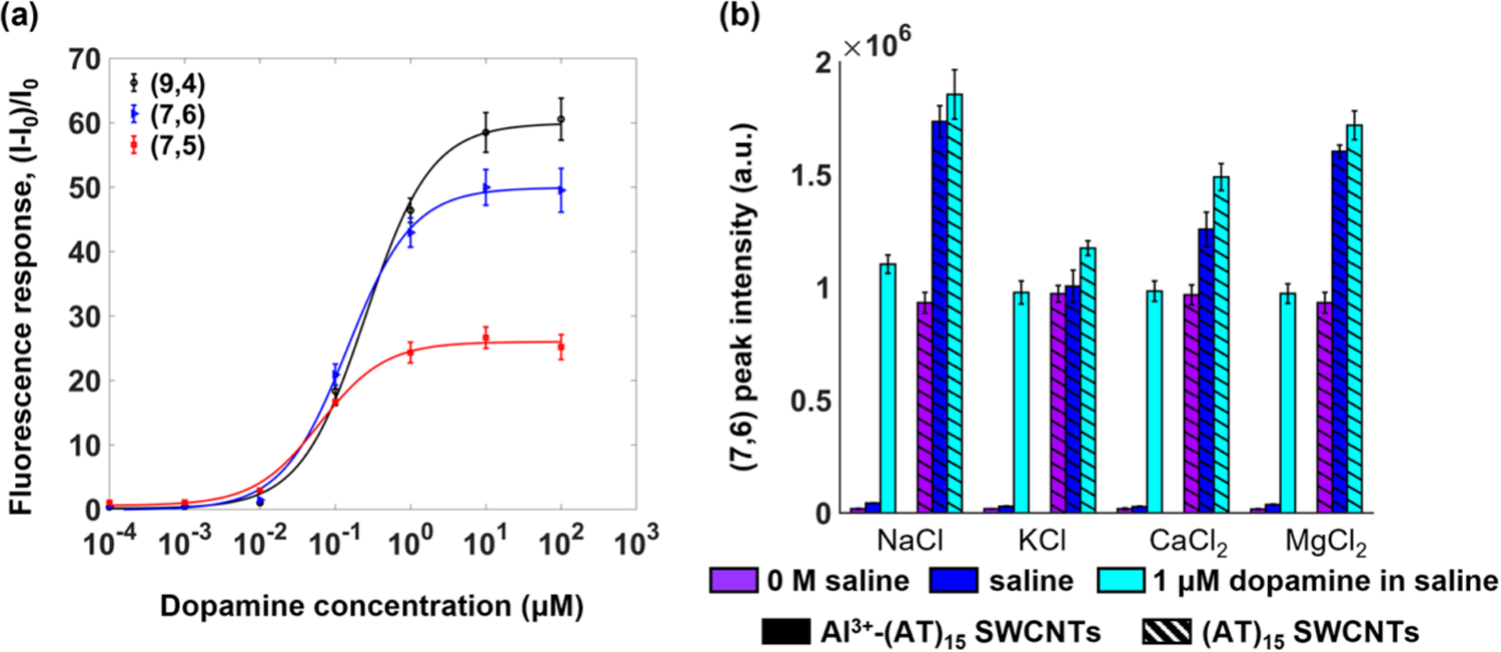
Effects of the conccentration and different salines on the dopamine response at pH 4.5. (a) Fluorescence response of the (9,4), (7,6), and (7,5) gel-encapsulated Al^3+^-pretreated (AT)_15_-SWCNTs to different dopamine concentrations at pH 4.5. Fluorescence response was calculated as the difference between the final intensity (I) and initial intensity (I_0_) divided by I_0_. Samples were excited at 655, 730, and 580 nm excitation for the (7,6), (9,4), and (7,5) chiralities, respectively, and emission intensities were measured at the corresponding chirality peak maximums. (b) Fluorescence intensity of (7,6) gel-encapsulated (AT)_15_-SWCNTs in the absence of salt (violet), in the presence of saline (blue), and after 10 mM dopamine addition in the presence of saline (cyan). Measurements were taken for (AT)_15_-SWCNTs pretreated with 1 M Al(NO_3_)_3_ (Al^3+^-(AT)_15_-SWCNTs, solid) and untreated (AT)_15_-SWCNTs ((AT)_15_-SWCNTs, patterned). The sensors were exposed to physiological saline concentrations of 150 mM NaCl, 10 mM KCl, 5 mM CaCl_2_, or 2 mM MgCl_2_ at pH 4.5. Samples were excited at 655 nm, and emission intensities were measured at the (7,6) chirality peak maximum. Error bars represent the standard deviation of three replicates.

This response was further studied by monitoring the fluorescence increase over a range of dopamine concentrations at a constant pH (Figure 5a). The measurements were taken at a pH of 4.5 to maximize the dopamine response while minimizing the effects of aluminum hydrolysis, which dominates at pH ≥ 5 (Figure S4). As shown in Figure 5a, the Al^3+^-treated (AT)_15_-SWCNTs show an increase in response to increasing dopamine. These observations are consistent with previous studies that have shown an increase in ssDNA-SWCNT fluorescence on dopamine addition.^6,49^ Although the redox potential has been shown to play a role in fluorescence increase,^49,50^ the dopamine addition results in no change in the SWCNT absorption.^49^ These findings indicate that the fluorescence brightening is unlikely to be due to charge transfer between the dopamine and SWCNT. Charge transfer between the dopamine and DNA is also unlikely, particularly since (AT)_15_ lacks the guanine that often serves as an electron acceptor.^6^ Molecular dynamics^46^ and measurements based on dye labeling of DNA^6^ have revealed, however, contributions from dopamine-induced changes in the DNA confirmation. These findings therefore suggest a mechanism that is based on DNA conformational changes, as opposed to static DNA interactions, consistent with the conclusions of previous studies.^45^

The dynamic range of this response varies with chirality, with the (7,5), (7,6), and (9,4) chiralities achieving dynamic responses between 0.1 and 10 μM, between 10 and 10 μM, and between 10 and 100 μM, respectively (Figure S5). At the maximum concentrations, the (9,4) and (7,6) chiralities show a saturating c.a. 60- and 50-fold response that exceeds the c.a. 1.25-fold maximum response of (GT)_15_-SWCNTs.^8^ The saturating response also exceeds the c.a. 35-fold response reported for previously engineered ssDNA-SWCNTs that were based on quenched baseline fluorescence.^48^ The expanded dynamic range of these sensors further aligns with the localized dopamine concentrations released from neurons, which vary from 0.01 μM to over 1 μM following stimulation.^47^ They also correspond to expected dopamine levels in urine, typically ranging from 65 to 400 μg (420 to 2612 nmol)^51^ per daily volume of 800–2000 mL,^52^ equivalent to concentrations between 0.2 and 3.3 μM dopamine. In addition to dynamic ranges, the chiralities show different sensitivities, with the (7,5) and (9,4) chiralities showing the lowest and highest Hill slopes at their corresponding half-maximal concentrations of 61.8 and 253.2 nM, respectively. The variations in sensitivity and dynamic range may stem from the uneven distribution of chiralities in the SWCNT mixture rather than differences in exciton dynamics or dopamine interactions. Nonetheless, these differences provide a valuable opportunity to optimize the sensors for specific measurement conditions and applications.

The performance of the Al^3+^-treated sensor was also compared with that of the untreated sensor in the presence of different saline solutions at pH 4.5 (Figure 5b). Body fluids such as blood, cerebrospinal fluid (CSF), and urine contain Na^+^, K^+^, Ca^2+^, and Mg^2+^ cations that may interfere with the SWCNT baseline fluorescence.^2,17^ As shown in Figure 5b, the addition of physiological concentrations of these salts, corresponding to 150 mM NaCl, 10 mM KCl, 5 mM CaCl_2_, and 2 mM MgCl_2_,^53^ does not affect the fluorescence of Al^3+^-treated (AT)_15_-SWCNTs. These findings are consistent with the Al^3+^ occupation of the DNA phosphate groups, which prevents further interactions between other cations and the phosphate groups. On the other hand, the untreated (AT)_15_-SWCNTs increase in fluorescence on the addition of all the cations except K^+^, which has a negligible effect under the tested conditions. On the addition of 1 μM dopamine, the Al^3+^-treated sensors show a drastic enhancement in fluorescence compared to the untreated sensors in all the cases. Except for the KCl, which yields a higher final intensity for the Al^3+^-treated sensor compared to the untreated sensor, the treated and untreated sensors show comparable final intensities following the dopamine addition. Since the 1 μM dopamine concentration falls below the saturation limit of the sensor (see Figure 5a), these findings suggest that the Al^3+^ does not necessarily enhance the efficacy of dopamine in reducing exciton quenching. Rather, the Al^3+^ appears to largely affect only the initial quenched state of the sensor and its resilience against interfering salt effects.

## Conclusions

The Al^3+^ pretreatment quenches the initial fluorescence of gel-immobilized (AT)_15_-SWCNTs, resulting in a lower fluorescence baseline for discerning a turn-on dopamine response. Though lower baselines have been previously achieved by modifying the DNA length,^48^ the pretreatment approach herein offers a generalizable strategy that relies on interactions with phosphate groups that are prevalent in all unmodified DNA. Furthermore, the increased quenching efficacy of the Al^3+^ achieves up to a ca. 70% enhancement in response compared to the sensor based on shortened DNA, establishing a new benchmark for SWCNT optical responses to dopamine.

This quenching is attributed to Al^3+^ binding of the phosphate background and charge withdrawal from the SWCNT surface. This mechanism is supported by the lack of quenching observed in surfactant-suspended SWCNTs,^24^ suggesting the direct metal-induced quenching of SWCNTs by Al^3+^ to be unlikely. Accordingly, Al^3+^ treatment occupies the binding sites of competing cations, minimizing the effects of interfering salts. Since dopamine addition does not alter SWCNT absorption,^49^ fluorescence brightening by charge transfer is also unlikely. These observations are consistent with a mechanism that increases fluorescence through alteration of DNA proximity to the SWCNT,^45^ as reported in previous studies.^45^ These findings thus suggest a mechanism that relies on DNA as a charge-based actuator that modulates fluorescence through charge withdrawal and conformational surface placement.

This sensing mechanism enables biological applications that require high signal-to-noise measurements such as spatiotemporal and single-molecule mapping of dopamine release from cells. Previous studies have relied on the direct immobilization of ssDNA-SWCNTs on the glass to monitor dopamine efflux from overlaid PC12 cells.^46^ In these measurements, the underlying ssDNA-SWCNTs showed a fluorescence increase in dopamine release. The application herein allows the cells to be grown atop washed gel-encapsulated SWCNTs that can isolate the effects of Al^3+^. These applications especially benefit from the turn-on response of a quenched fluorescence baseline, which increases the signal-to-noise ratio and decreases the uncertainty in these biological measurements.

## Materials and Methods

### Preparation of ssDNA-Suspended SWCNTs

The ssDNA sequences (AT)_15_ and (GT)_15_ (Microsynth) were dissolved in deionized water (18.2 MΩ·cm) to a final concentration of 100 μM. The SWCNTs (NanoIntegris, lots HP26-019 and HP29-064) were added to the ssDNA solution to yield a 1 mg/mL mixture with a 1:1 SWCNT:ssDNA mass ratio. The mixture was sonicated at 4 °C in a cuphorn sonicator for 90 min at 1% amplitude (Q700, QSonica). The resulting suspension was centrifuged at 4 °C for 4 h (Eppendorf Centrifuge 5425R), and ca. 80% of the supernatant containing the ssDNA-SWCNT suspension was collected for further treatment. The remaining unbound ssDNA in this suspension was removed through centrifugal filtration (Amicon Ultra 0.5 100 kDa). The purified ssDNA-SWCNT suspension was then diluted to an optical density of 0.742 at 632 nm.

### Preparation of ssDNA-SWCNT Gel

Agarose (Sigma-Aldrich) was dissolved in deionized water to yield a 2 wt % solution. The ssDNA-SWCNT and agarose gel solutions were mixed in a 1:1 volume ratio, and 190 μL of this mixture was added to the bottom of a glass-bottomed Petri dish. The gel was dried for at least 10 min. After solidifying, the gel was incubated in deionized water for 4 h to stabilize the SWCNT fluorescence.

### Preparation of Ion-Bound ssDNA-SWCNT Gel

The gel was immersed in either 1 M NaCl, 1 M MgCl_2_, 1 M CaCl_2_, or 1 M Al(NO_3_)_3_ (Sigma-Aldrich) solution. After 2 h of incubation, the gel and Petri dish were washed with deionized water. The gel was then immersed in 1 L of HCl solution (pH 3.7) to remove the unbound ions, and the solution was discarded after 2 h of incubation. This HCl washing step was repeated 4 additional times.

### SWCNT Fluorescence Measurements

SWCNT fluorescence measurements were taken with a custom-built near-infrared inverted microscope described previously^54^. The Petri dishes were excited at 655 nm for (7,6) chirality measurements, 730 nm for (9,4) chirality measurements, and 580 nm for (7,5) chirality with an exposure time of 5 s. Emissions were processed with a spectrometer (IsoPlane SCT-320, Princeton Instruments) and collected with an InGaAs camera (NIRvana 640 ST, Princeton Instruments). The measurements were plotted by using a custom Matlab code. The wavelength emissions were determined based on the maximum peak intensity. Changes in peak intensities were calculated based on the difference at the maximum.

## Supporting Information Available

Protocol for sensor preparation; calculation of effective Al^3+^ concentration in gel; fitting method for Figure 5a; emission spectra of (AT)_15_-SWCNTs before and after Al^3+^ incubation; fluorescence intensity and peak wavelength of (7,5) gel-encapsulated (AT)_15_-SWCNTs over time following the addition of 1 M Al(NO_3_)_3_; intensity responses of (9,4) gel-encapsulated (AT)_15_-SWCNTs; emission spectra of Al^3+^-(AT)_15_-SWCNTs before and after 100 μM dopamine addition at pH 4.5; fluorescence intensities of the (7,6), (9,4), and (7,5) gel-encapsulated Al^3+^-pretreated (AT)_15_-SWCNTs to different dopamine concentrations at pH 4.5 (PDF)
