# Supplementary material for: Cation Pretreatment Enables the Saline Stability of a Near-Infrared Sensor for Dopamine
Source: ACS Bio Med Chem Au. 2025 Jan 27;5(1):166–74. doi: 10.1021/acsbiomedchemau.4c00094 (PMC11843333; doi:10.1021/acsbiomedchemau.4c00094)
Supplement: Supplementary file 1 — bg4c00094_si_001.pdf [file bg4c00094_si_001.pdf]

Supporting Information

Cation Pretreatment Enables the Saline Stability of a Near-Infrared Sensor for Dopamine

Xuewen Liu[a], Jing Chen[a], Hanxuan Wang[b], Benjamin Lambert[c], Ardemis A. Boghossian[b]

[a]Henan Agricultural University, Zhengzhou, Henan, 450002, China
[b]Ecole Polytechnique Fédérale de Lausanne (EPFL), 1015 Lausanne, Switzerland
[c]Université de Bordeaux, LP2N - Institut d'Optique, CNRS, F-33405 Talence, France

Protocol for sensor preparation:

(1) Preparation of ssDNA- suspended SWCNTs

The $(AT)_{15}$ and $(GT)_{15}$ ssDNA sequences were ordered from Microsynth. The single-walled carbon nanotubes (SWCNTs) were ordered from NanoIntegris (Lot No. HP26-019 and HP29-064).

- Dissolve 100 μM the ssDNA in deionized water (18.2 MΩ·cm resistivity).
- Add 1 mg/mL SWCNTs (1:1 mass ratio of SWCNTs to ssDNA).
- Mix the ssDNA and SWCNT solution.
- Suspend the SWCNTs by sonicating for 90 minutes at 1% amplitude (cuphorn, Q700, QSonica) at a controlled temperature of 4°C.
- Centrifuge the suspension for 4 hours at 4 °C (Eppendorf Centrifuge 5424R) and collect 80% of the supernatant.
- Remove the unbound ssDNA sequences in the supernatant with Amicon Ultra 0.5 100 kDa centrifugal filters.
- Dilute the ssDNA-SWCNT suspension to a final absorbance of 0.742 at 632nm.

(2) Preparation of ssDNA-SWCNT gels

- Prepare 2% agarose gel with deionized water.
- Mix the ssDNA-SWCNT solution and agarose gel with a 1:1 volume ratio.
- Add 190 μl of the above gel mixture to the petri dish.
- Wait for at least 10 minutes, until the ssDNA-SWCNT gel solidifies.
- Incubate the solidified gel in deionized water for 4 hours to stabilize the fluorescence.

(3) Preparation of Al^{3+}-stabilized ssDNA-SWCNT gels

- Add 1 M $Al(NO_3)_3$ solution into the petri dish and incubate for 2 hours.
- Discard the used $Al(NO_3)_3$ solution, and wash the petri dish and gel with deionized water.
- Immerse the gel in 1 L HCl solution (pH 3.7) for 2 hours to remove the free Al^{3+} ions that remain in the gel.
- Repeat the immersion 4 times to remove any remaining unbound Al^{3+}.

<u>Calculation of effective Al^{3+} concentration in gel</u>

Mass of SWCNTs per sensor:

As specified in the methods, the final $(AT)_{15}$-SWCNT solution was adjusted to an absorbance of 0.742 at 632 nm (A_{632}[illegible] = 0.742). Assuming an extinction coefficient of ε_{632}[illegible] = 0.036 L mg^{-1} cm^{-1},[1] for a cuvette with a path length of b = 1 cm, the concentration, c, of SWCNTs is calculated as

$c = A_{632}$[illegible] $/(\varepsilon_{632}$[illegible] $\times b) = 0.742 / (0.036\ L\ mg^{-1}\ cm^{-1} \times 1\ cm)$
$= 20.611\ mg/L = 2.06 \times 10^{-5}\ mg/\mu L$

On 1:1 mixing with the agarose solution, the agarose mixture concentration, $c_{agarose}$, is a 1:2 dilution of the original mixture,

$c_{agarose} = (2.06 \times 10^{-5}\ mg/\mu L) / 2$
$= 1.03 \times 10^{-5}\ mg/\mu L$

Drop-casting a volume of V = 190 µL = 1.9 × 10^{-4} L of this mixture per sensor, the total mass of SWCNTs per sensor, m_{SWCNTs}, is calculated as

$m_{SWCNTs} = c_{agarose} \times V = 1.03 \times 10^{-5}\ mg/\mu L \times 190\ \mu L$
$= 0.00196\ mg = 1.96 \times 10^{-6}\ g$

Number of SWCNTs per sensor:

The number of SWCNTs per sensor can be calculated from the molecular weight of the SWCNT. The SWCNT mixture consists of a distribution of SWCNT chiralities and lengths. For an average SWCNT length of L_{SWCNT} = 600 nm [2] and average diameter of 0.8, which is comparable to the 0.83 average diameter of a (7,6)-enriched mixture (product description of Sigma Aldrich Product 704121, Carbon nanotube, single-walled), one can assume 80 carbon atoms per 0.283 nm length of SWCNT (C_{nm} = 282.69 C atoms/nm) (product description of Raymor HiPCO Single Wall Carbon Nanotubes). Given the molecular weight of carbon, MW_C, of 12.01 g/mol, the molecular weight of the SWCNTs, MW_{SWCNTs}, is approximated as

$MW_{SWCNTs} = C_{nm} \times L_{SWCNT} \times MW_C = 282.69\ C\ atoms/nm \times 600\ nm \times 12.01\ g/mol$
$=2.04 \times 10^{6}\ g/mol$

The number of SWCNTs, N_{SWCNTs}, per sensor is calculated by dividing the mass of SWCNTs, m_{SWCNTs}, by the molecular weight, MW_{SWCNTs}, and multiplying by Avogadro's number, N_A,

$N_{SWCNTs} = m_{SWCNTs} / MW_{SWCNTs} \times N_A = 1.96 \times 10^{-6}\ g / (2.04 \times 10^{6}\ g/mol) \times 6.02 \times 10^{23}$ molecules/mol
$= 5.79 \times 10^{11}$ molecules SWCNT

Effective concentration of Al^{3+} in sensing gel:

Approximately 170 molecules of the $(AT)_{15}$ ssDNA sequence are adsorbed per 600 nm-long SWCNT ($N_{DNA/SWCNT}$ = 170 molecules ssDNA/molecule SWCNT).[2] The total number of ssDNA molecules per sensing gel, N_{ssDNA}, is calculated as the product of the number of molecules of ssDNA per SWCNT, $N_{DNA/SWCNT}$, and the total number of SWCNTs, N_{SWCNTs},

$N_{ssDNA} = N_{DNA/SWCNT} \times N_{SWCNTs}$ = 170 molecules ssDNA/molecule SWCNT × (5.79 × 10^{11} molecules SWCNT)
= 9.84 × 10^{13} molecules ssDNA

Assuming approximately 5 Al^{3+} bound cations for each of the 30-bp ssDNA molecules ($N_{Al3+/ssDNA}$ = 5 molecules Al^{3+}/ssDNA) [3] the total number of bound Al^{3+} cations in the gel, $N_{Al^{3+}}$, can be calculated as the product of the number of bound cations per ssDNA, $N_{Al^{3+}/ssDNA}$, and the total number of ssDNA, N_{ssDNA},

$N_{Al^{3+}} = N_{Al^{3+}/ssDNA} \times N_{ssDNA}$ = 5 molecules Al^{3+}/molecule ssDNA × 9.84 x 10^{13} molecules ssDNA
= 4.92 × 10^{14} molecules Al^{3+}

The effective concentration, c_{Al3+}, can be calculated by dividing the number of molecules of Al^{3+}, $N_{Al^{3+}}$, by Avogrado's number, N_A, to determine the number of moles, and then dividing by the volume, V,

$c_{Al3+} = (N_{Al^{3+}} / N_A)/ V$ = [4.92 × 10^{14} molecules Al^{3+}/ (6.02 × 10^{23} molecules/mol)] / (1.9 × 10^{-4} L)
= 4.30 × 10^{-6} mol/L = 4.30 × 10^{-6} M = 4.30 µM

The corresponding log value used for the phase diagram is

$\log(c_{Al^{3+}}) = \log(4.3 \times 10^{-6}\ M) = -5.37$

Response curve fitting for Figure 5a

The concentration-response profile in Figure 5a was fit using the 'dose-response inhibition' function of the GraphPad Prism software. The fitting curve was calculated based on the equation,

$$Y = Bottom + \frac{Top - Bottom}{1 + 10^{(\mathrm{LogIC50} - X)}}$$

where *Top* represents the magnitude of the maximum sensor response, and *Bottom* represents the minimum sensor response. The benchmark response (BMR) was defined as 10% to 90% of the maximum response of the (7,5) chirality, and the corresponding concentrations at these responses were used to define the dynamic range of concentrations that elicit an effective sensor response.

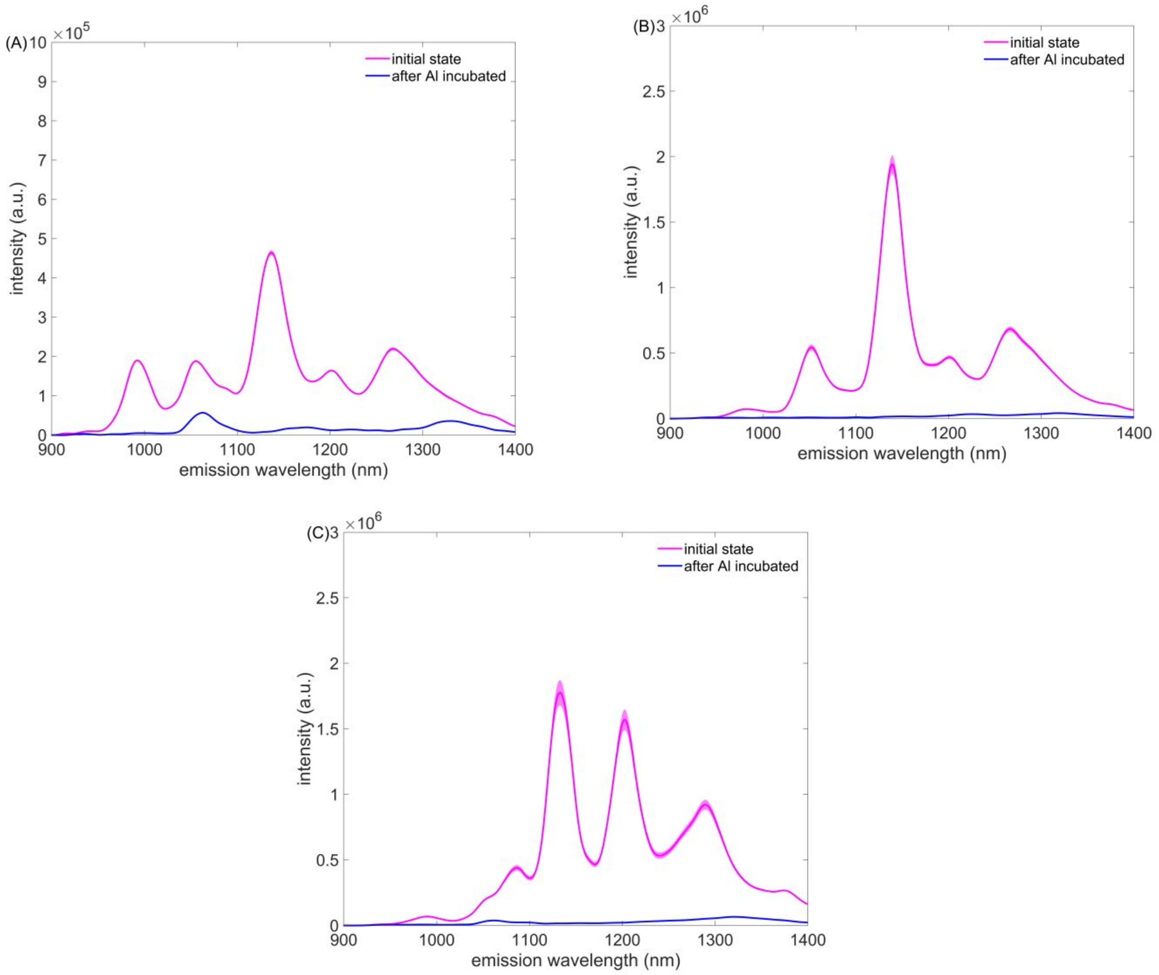

Figure S1. Emission spectra of $(AT)_{15}$-SWCNTs before (pink) and after (blue) Al^{3+} incubation with **(A)** 580 nm, **(B)** 655 nm, and **(C)** 730 nm excitation.

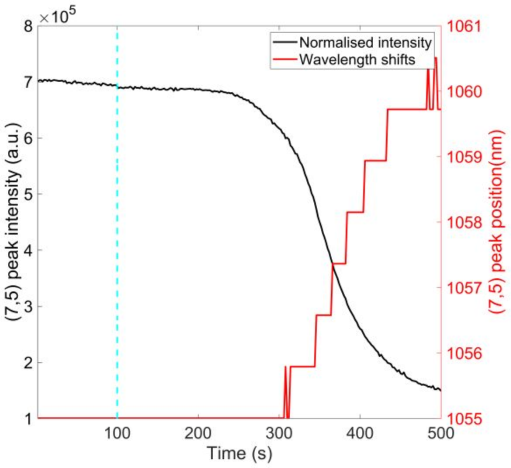

Figure S2. Fluorescence intensity (black) and peak wavelength (red) of (7,5) gel-encapsulated $(AT)_{15}$-SWCNTs over time following the addition of 1 M $Al(NO_3)_3$. Samples were excited at 650 nm and emission intensities were measured at the (7,5) chirality peak maximum. The cyan vertical dotted line corresponds to the time at which $Al(NO_3)_3$ was added.

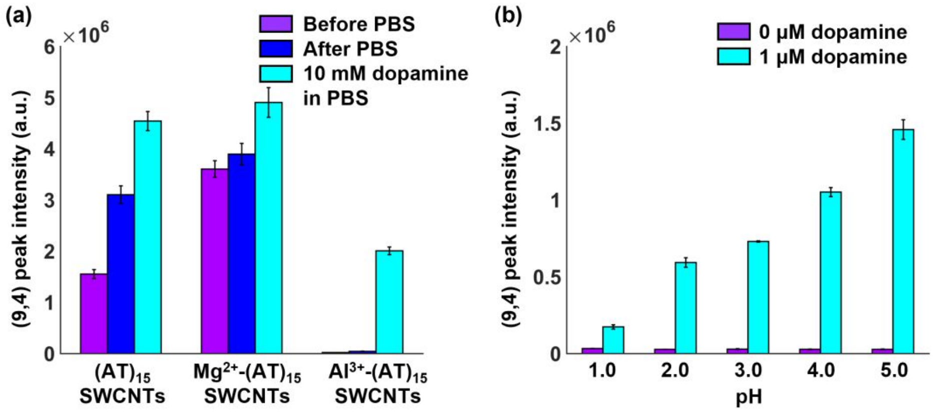

Figure S3. (a) Fluorescence intensity of (9,4) gel-encapsulated $(AT)_{15}$-SWCNTs in the absence of buffer (violet), in the presence of PBS buffer (blue), and after 10 mM dopamine addition in the presence of buffer (cyan). Measurements were taken for untreated $(AT)_{15}$-SWCNTs ($(AT)_{15}$-SWCNTs, left), $(AT)_{15}$-SWCNTs pretreated with 1 M $MgCl_2$ (Mg^{2+}-$(AT)_{15}$-SWCNTs, center), and $(AT)_{15}$-SWCNTs pretreated with 1 M $Al(NO_3)_3$ (Al^{3+}-$(AT)_{15}$-SWCNTs, right). **(b)** Fluorescence intensity of (9,4) gel-encapsulated Al^{3+}-pretreated $(AT)_{15}$-SWCNTs at different pHs in the absence (violet) and presence (blue) of 1 μM dopamine.

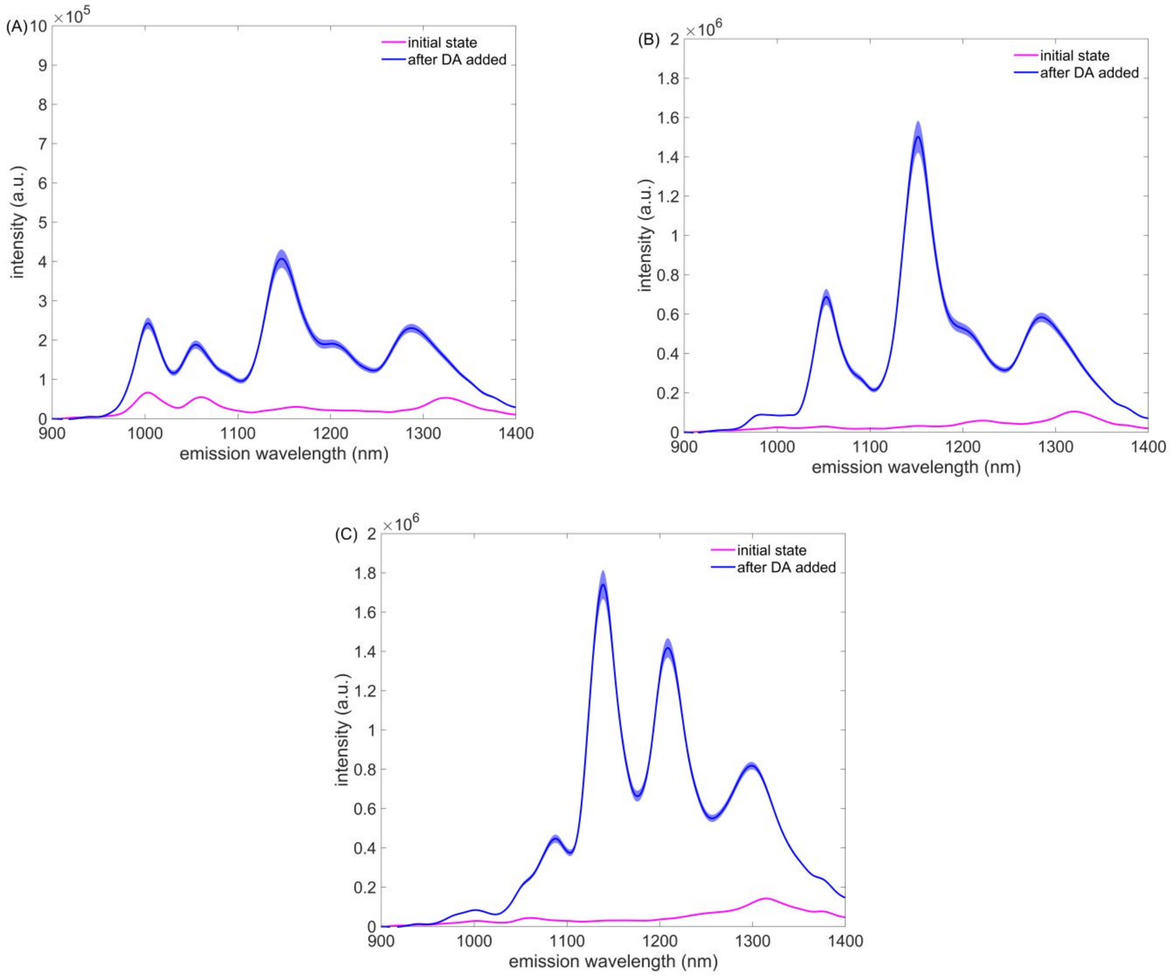

Figure S4. Emission spectra of Al^{3+}-$(AT)_{15}$-SWCNTs before (pink) and after (blue) 100 µM dopamine addition at pH 4.5 with **(A)** 580 nm, **(B)** 655 nm, and **(C)** 730 nm excitation.

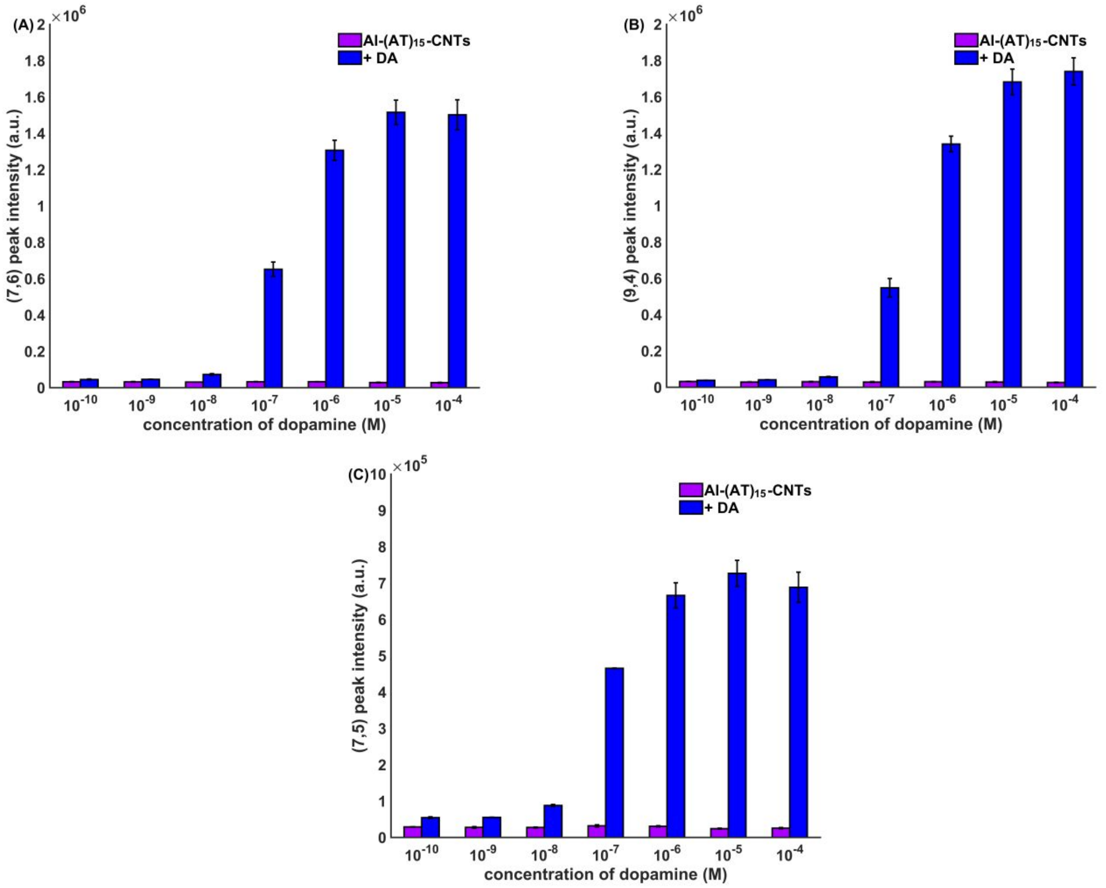

Figure S5. Fluorescence intensities of the **(a)** (7,6), **(b)** (9,4), and **(c)** (7,5) gel-encapsulated Al^{3+}-pretreated $(AT)_{15}$-SWCNTs to different dopamine concentrations at pH 4.5. Samples were excited at 655 nm, 730 nm, and 580 nm excitation for the (7,6), (9,4), and (7,5) chiralities, respectively, and emission intensities were measured at the corresponding chirality peak maximums

Reference

(1) Zhang, J., et al., *A rapid, direct, quantitative, and label-free detector of cardiac biomarker troponin T using near-infrared fluorescent single-walled carbon nanotube sensors.* Adv Healthc Mater, 2014. **3**(3): 412-423.
(2) Nißler, R., et al., *Quantification of the Number of Adsorbed DNA Molecules on Single-Walled Carbon Nanotubes.* The Journal of Physical Chemistry C, 2019. **123**(8): 4837-4847.
(3) Andresen, K., et al., *Mono- and trivalent ions around DNA: a small-angle scattering study of competition and interactions.* Biophys J, 2008. **95**(1): 287-295.
